# Electrocardiographic parameters and mortality in patients with SARS-CoV-2 infection: A single center study

**DOI:** 10.22088/cjim.15.3.444

**Published:** 2024-08-01

**Authors:** Samaneh Karbalaie Esmaeilzad, Mohammad Taghi Hedayati, Naghmeh Ziaie, Hoda Shirafkan, Farzad Jalali, Iraj Jafaripoor, Kamyar Amin, Rogheyeh Pourkia, Zahra Jabbary, Saeid Abroutan, Mehrdad Saravi

**Affiliations:** 1 School of Medicine, Babol University of Medical sciences, Babol, Iran; 2 Clinical Research Development Unit of Ayatollah Rouhani Hospital, Babol University of Medical Sciences, Babol, Iran; 3 Department of Cardiology, School of Medicine, Babol University of Medical Sciences, Babol, Iran; 4 Social Determinants of Health Research Center, Health Research Institute, Babol University of Medical Sciences, Babol, Iran

**Keywords:** Electrocardiogram, SARS-CoV-2 infection, Mortality

## Abstract

**Background:**

Coronavirus disease 2019 (COVID-19) is a pandemic outbreak of RNA coronaviruses (SARS-CoV-2), associated with acute respiratory distress syndrome, multiple organ failure, and death. The surface electrocardiogram is the first line assessment of cardiac electrical system. We aimed to interpret classically the electrocardiographic parameters at admission and during hospital course and association of them with prognosis in patients admitted with diagnosis of infection with SARS-CoV-2.

**Methods:**

Surface electrocardiograms (ECG) were obtained from 180 patients with SARS-CoV-2 infection at a large tertiary referral university hospital at north of Iran in Babol. The electrocardiographic waves, intervals and segments in addition to supraventricular and ventricular arrhythmias were depicted. Our cohort included two groups: discharged alive and dead during the hospital course. We compared the ECG characteristics of patients who died vs. survived ones.

**Results:**

Some ECG parameters of 180 hospitalized patients were significantly associated with mortality, like heart rate (p< 0.001), bundle branch block (P= 0.035), fragmented QRS (P= 0.015), ST elevation (P= 0.004), T p-e duration (P= 0.006), premature atrial and ventricular complexes (P= 0.030, P= 0.004) and atrial fibrillation (P= 0.003).

**Conclusion:**

The SARS-CoV-2 infection had several impacts on cardiac electrical system which may monitored with a simple and easily accessible tool like ECG. This tool also helpful in the risk stratification of patients.

Severe acute respiratory syndrome coronavirus 2 (SARS-CoV-2), the pathogen responsible for coronavirus disease 2019 (COVID-19), initially identified as primarily affecting the respiratory system, has now emerged as a complex systemic illness with significant implications for cardiovascular health (1). Prompt identification of individuals predisposed to adverse outcomes is imperative for effective patient management. Electrocardiograms (ECGs) represent a readily accessible and non-invasive modality for evaluating cardiac function (2). Numerous investigations have sought to elucidate the spectrum of ECG abnormalities and their correlation with clinical outcomes, aiming to elucidate the intricate interplay between SARS-CoV-2 infection and cardiovascular sequelae. Increasing evidence suggests that SARS-CoV-2 infection can induce a range of ECG alterations, from nonspecific ST-T wave changes to potentially life-threatening arrhythmias (3). While the precise mechanisms remain under investigation, viral pathogenicity, systemic inflammation, hypoxia, and endothelial dysfunction are implicated.

Prior studies have highlighted associations between abnormal ECG findings in COVID-19 patients and heightened risks of mortality, intensive care admission, and cardiovascular complications.

However, these investigations often encompass diverse patient cohorts across multiple healthcare settings, introducing variability in demographics, treatment modalities, and ECG interpretation practices (4). Additional research is warranted to clarify the relationship between specific electrocardiographic markers and mortality risk in SARS-CoV-2 patients. 

In this study, conducted at a single center, we aim to elucidate the association between distinct ECG parameters and in-hospital mortality among individuals hospitalized with SARS-CoV-2 infection (5, 6). Through comprehensive analysis of a diverse patient cohort, we endeavor to delineate the prevalence of ECG abnormalities and their prognostic relevance. Our findings hold promise for enhancing comprehension of cardiac involvement in COVID-19 and exploring the utility of ECG as a prognostic tool for risk stratification in this vulnerable population. By integrating ECG data with clinical outcomes, our investigation aims to furnish clinicians with refined prognostic tools for early risk identification and personalized therapeutic interventions, thereby ameliorating patient care and outcomes amidst this global health crisis. As we navigate the complex landscape of COVID-19-related cardiovascular complications, our study endeavors to enrich the evidence base informing clinical decision-making and public health strategies.

## Methods

**Study design and participants:** Our inpatient cohort consisted of 180 patients enrolled at this prospective observational single center (Ayatollah Rouhani Hospital, Babol University of Medical Sciences, Babol, Iran) study from April 2020 to 2021.The diagnosis was based on the World Health Organization’s guidance (7), which was confirmed by a nasopharyngeal swab for a reverse transcription-polymerase chain reaction. Patients more than 18 years old, who agreed to participate and signed informed consent, and underwent standard ECG recording at admission and in-hospital course were included. The exclusion criteria were pacemaker rhythm and ventricular pre-excitation on ECG. We systemically evaluated all clinical records and detailed medical history for any demographic characteristics, medications, laboratory data, and preexisting co-morbidities like coronary artery disease (documented obstructed coronary arteries, old myocardial infarction, or any coronary revascularization), heart failure, stroke (in recent 24 months), malignancy, chronic obstructive pulmonary disease (Gold stage III, IV) and renal failure (stages 4-5). The present study was approved by the local Ethics Committee IR.MUBABOL.HRI.REC.1400.006

**Electrocardiography analysis and definitions: **A standard 12-lead ECG was recorded at 25 mm / s, 1 cm calibration, and a filter setting of 0.05 Hz, using a Mortara 150 device (Mortara Machine, Bologna, Italy). The analyses performed by two fellowships of cardiac electrophysiology (MN, M.M.) who were unaware of the details of the study. The discrepancy was resolved by the surveillance and consensus of the third one. All arrhythmias during hospitalization were also recorded. The following parameters were outlined: heart rate, rhythm, QRS width and bundle branch blocks, and atrioventricular blocks, ST segment elevation (J point elevation of ≥1 mm, ST-segment elevation > 80 ms with morphology in favor of pericardial or coronary involvement. Patients with left bundle branch block pattern and leads V1-4 in right bundle branch block pattern were excluded from ST-segment and T wave analyses. T wave inversion (≥1 mm in at least two contiguous leads except aVR and V1) and from park to end of T wave (Tp-e) were revealed. 

Bazett's formula [QTc=QT (ms) /√RR (s) ] was used for calculating corrected QT interval, but in wide QRS complexes (≥ 120 ms), we used recommendations of Bogossian H, et al. (8) Premature atrial and ventricular complexes (PAC, PVC respectively) , atrial fibrillation, atrial flutter, ventricular tachycardia, ventricular fibrillation were detailed. 

**Statistical analysis:** All data were analyzed using SPSS Version 25.0 (IBM Corporation). Normally distributed continuous variables were expressed as mean±standard deviation (S.D.), and categorical variables were expressed as counts and percentages. We checked normality of data, then t-test and Mann-Whitney U test were used for comparing the mean of variables in two groups of dead and alive patients. Chi-square and Fisher s exact test was used for assessing categorical variables. Univariate and Multivariate regression analyses were performed for association of clinical, laboratory, and ECG parameters with mortality. The findings were reported as odds ratio (OR) and 95% confidence interval (95% CI). The p-values ​​less than 0.05 for was considered statistically significant. 

## Results

**Population characteristics:** From 10 April 2020-2021, one hundred-eighty patients were enrolled to our cohort with positive PCR for SRAS-CoV-2 admitted in Ayatollah Rouhani Hospital (Babol University of Medical Sciences, Babol, Iran) who divided in two equal age and sex-matched groups of alive and dead. Baseline patient characteristics were detailed in table 1. The mean age of the entire cohort was 64.4 ± 12.51 years (range: 34-89 years). Males were slightly more common than females (101 [56.1%] vs 79 [43.9%]. Ninety patients died in hospital course. The most common comorbidities were hypertension (32.8%) and diabetes (20.6%). They had a history of coronary artery disease (15%), heart failure (7.8%), and chronic obstructive pulmonary disease (5%). The incidence of abnormal amounts of cTnI, NT-proBNP and hs-CRP (p < 0.004, p < 0.004 and p< .005 respectively) were higher in patients who died than others (table 1). 

**Table 1:** Baseline patients’ characteristics of patients infected with SARS-CoV infection

	**All (n=180)**	**Survived (n- 90)**	**Died (N=90)**	**P value**	**Odds ratio**	**95% CI**
**Age (Years)**	64.4 ± 12.51	63.69 ± 11.05	64.40 ± 13.87	0.704	1.005	0.981-1.028
**Sex (F/M)**	79 (43.9%)	39(43.3)	40 (44.4)	0.881	0.956	0.53-1.72
**CAD**	27 (15%)	13 (14.4%)	14 (15.6%)	0.835	1.091	0.481-2.474
**Heart Failure**	14 (7.8%)	8 (8.9%)	6 (6.7%)	0.002	1.091	0.481-2.474
**Hypertension**	59 (32.8%)	24 (26.7%)	35 (38.9)	0.082	1.750	0.931-3.288
**Diabetes mellitus**	37 (20.6%)	15 (16.7%)	22 (24.4%)	0.03	1.618	0.777-3.369
**Smoking**	45	19 (21.1%)	26 (28.9%)	0.230	1.518	0.768-3.000
**COPD**	9 (5%)	4 (4.4%)	5 (5.5%)	0.346	1.577	0.612-4.064
**C-reactive protein, mg/L**	59.59 ± 36.37	42.04 ± 16.09	77.14 ± 42.17	0.005	1.064	1.041-1.87
**Elevated cTNI ng/L**	65 (36.1%)	7 (7.8%)	58 (64.4%)	0.004	21.491	8.879-52.017
**Elevated NT-proBNP**	32 (17.8%)	26 (28.9%)	6 (6.7%)	0.004	5.687	2.210-14.638

CI Confidence Interval, F female, M male, CAD coronary artery disease, COPD chronic obstructive pulmonary disease, cTNI cardiac troponin I, NT-proBNP N-terminal pro-brain natriuretic factor.

Electrocardiographic findings: The main electrocardiographic findings were summarized in table 2. Most (83%) patients had sinus rhythm on ECG. Abnormal ECGs were observed in nearly all patients who died, compared with 63% of patients who survived. Baseline ECG values ​​included mean heart rate was higher in expired group than survived one (88.63 ± 26.32 vs. 77.07 ± 11.61, P < 0.001, respectively), but mean PR interval was not different significantly between two groups (P=0.653). The average QRS duration was 87.39 ±32.45 msec. Patients with bundle branch blocks and fragmented QRS were seen more in patients who expired (p values 0.035 and 0.015, respectively).The mean Bazett-corrected QT interval was 389.09 ± 55.52 ms (range 370-693 ms). A corrected QT interval > 460 ms was observed in 25% of patients and with worse outcome ones (P=0.002). Local ST elevation and pathological T wave inversion were seen in 15 (8.3%) and 82 (45.6%) patients respectively, which had significant associated with poor outcome (P=0.004 and 0.03 respectively).

The interval which is an important index of depolarization was abnormal (P=0.006). Atrial fibrillation / flutter, atrioventricular block and abnormal intraventricular conduction was seen in 15%, 6.1% and 11.1% of patients respectively. Premature atrial and ventricular complexes occurred in 63.3% and 35% of participants respectively. Tachycardia was more common among those who died (88.63 ± 26.32 vs. 77.07 ± 11.61, p <0.001). 

**Table 2:** Electrocardiographic characteristics of 180 patients infected with SARS-CoV-2 infection

**Variable**	**All (n=180)**	**Survived (n- 90)**	**Died (N=90)**	**P-value**	**Odds ratio**	**95%CI**
**Heart rate**	84.35 ± 21.56	77.07 ± 11.61	88.63 ± 26.32	<0.001	1.038	1.020-1.056
**PR interval ms**	183.04 ± 34.12	171.67 ± 37.03	187.44 ± 47.6	0.653	0.983	1.018-1.049
**QRS, ms**	87.32 ± 21.12	85.56 ± 31.78	90 ± 38.06	0.601	0.993	2.143-3-187
**BBB**	20 (11.1)	6 (6.7%)	14 (15.6%)	0.035	2.579	0.944-7.049
**Fragmented QRS**	21 (11.7%)	5 (5.6%)	16 (17.8%)	0.015	3.676	1.284-10.519
**QTc interval**	389.09 ± 55.52	425.69 ± 36.28	452.68± 61.56	0.005	1.011	1.005-1.018
**Abnormal QTc**	135 (75%)	77 (85.6%)	58 (64.4%)	0.002	3.268	1.576-6.775
**ST-segment elevation**	15 (8.3%)	3 (3.3%)	12 (13.3%)	0.004	4.462	1.214-16.396
**T wave inversion**	82 (45.6%)	26 928.9%)	56 (62.2%)	0.03	4.54	2.172-7.567
**Tp-e ms**	72.98 ± 6.93	70.60 ± 5.40	75.36 ± 7.49	0.006	1.123	1.576-6.775
**Atrial Fibrillation/Flutter**	27 (15%)	6 (6.7%)	21 (23.3%)	0.003	4.261	1.629-11.145
**AV Blocks**	11 (6.1%)	3 (3.3%)	8 (8.9%)	0.134	1.829	0.726-11.032
**PVCs**	63 (35%)	16 (17.8%)	48 (53.3%)	0.004	5.286	2.676-10.442
**PACs**	114 (63.3)	35 (38.9%)	79 (87.7%)	0.030	11.286	5.278-24.1130

SD, Standard deviation, QTC, Corrected QT interval by Bazett’s formula, PAC, Premature Atrial Contractions, AVB Atrioventricular block, RBBB, Right bundle branch block, LBBB, Left bundle branch block, LAHB, Left anterior hemiblock, LPHB, left posterior hemiblock, LVH, left ventricular hypertrophy, RVH, right ventricular hypertrophy

**Table 3:** Multivariate logistic regression analysis of mortality in 180 SARS-CoV-2 infection infected patients with SARS-CoV-2

**Variable**	**P-value**	**Odds Ratio**	**95% CI**
**Heart rate bpm**	<0.001	1.025	1.011-1.042
**ST segment elevation(Yes/No)**	0.006	3.91	1.619-16.895
**Fragmented QRS (Yes/No)**	0.023	3.212	1.012-10.739
**Abnormal QTc interval ms**	<0.001	3.353	1.674-6.692
**T p-e index**	<0.001	1.258	1.085-1.228
**PAC (Yes/No)**	<0.001	12.152	6.645-25.048
**PVC (Yes/No)**	<0.001	5.877	3.283-10.739
**Atrial fibrillation or flutter (Yes/No)**	0.029	3.981	1.532-10.624

Bpm beat per minute, T p-e T peak-end interval, PAC premature atrial complex, PVC premature ventricular complex

## Discussion

Our results demonstrated that surface electrocardiography, as a simple and readily available tool, is useful in predicting risk stratification and clinical outcome in patients hospitalized with infection, especially in univariate and multivariate analyses. ECG variables independently associated with mortality were heart rate, ST-segment elevation, fragmented QRS, abnormal corrected QT interval, Tp-e interval, and atrial fibrillation. Clinical variables such as hypertension, diabetes, heart failure, and laboratory findings such as abnormal levels of troponin I, N-terminal proBNP, and C-reactive protein are associated with increased hospitalization complications and mortality due to CoV-19 infection. We found an association between hypertension, diabetes and heart failure as co-morbidities with mortality in infected ones with COVID-19. Zylla MM., et al. showed older age and cardiovascular risk factors predisposed for arrhythmia during hospitalization (9). Some researches (10-11). It suggests a significant association between clinical comorbidities such as diabetes, hypertension, obesity and smoking and disease severity. Sinus tachycardia is associated with increased risk, consistent with previous studies (12, 13). There was an association between QRS width prolongation and various types of bundle branch block with adverse outcomes (12-15). In univariate analysis, like Wang D et al.’s study (16, bradyarrhythmia, particularly AV conduction block, were associated with poor outcome. We showed that a fragmented QRS can predict mortality in patients like Yildirim A. et al. (17). 

Our results highlight that ST elevation is associated with an almost four-fold increase in mortality. This electrocardiogram change indicates myocardial tissue damage due to coronary artery stenosis, hypoxia, hemodynamic disturbances, and infarction due to myocarditis. (18, 19, 20). According to association between coronary risk factors (10, 20) such as hypertension and diabetes and increase mortality risk, ST elevation is associated with elevated troponin I and myocardial infarction (11). Abnormal T inversion and prolonged Tp-e interval was associated with (21, 22). This repolarization changes may be due to myocarditis, sub-endocardial ischemia-infarction and drug effects and associated with increase in arrhythmia risk profile (24) and mortality. We found an association between corrected QT interval prolongation and mortality. It is a marker of susceptibility to ventricular arrhythmias and death. Changes in the QT interval result from ischemia, inflammation, hemodynamic disturbances, and drug-induced myocardial damage. (26). Banaie A, et al. found association with disease severity, myocardial damage and one-year mortality in hospitalized patients with COVID-19 infection (27). Atrial fibrillation (AF) was observed in about one-sixth of our patients which was associated with poor prognosis including independent mortality risk. It is a risk factor for heart failure and stroke. Bhatla A et al. (28) reported association of AF (as the most common arrhythmia) and ICU admission and mortality (not independent factor in multivariate analysis). Mountantonakis S et al. showed a 17.6 % incidence of atrial fibrillation, especially new episodes, which was an independent predictor of in-hospital mortality (29). Paris S et al. in a multi-center study showed that history of AF was associated with higher mortality and in hospital events (30). The incidence of arrhythmias in Yunidai et al.’s study was 22%. Among them, atrial fibrillation was the most common one (12). Similar to previous studies (10, 31), increasing levels of biomarkers such as N-terminal pro-BNP, troponin I, and C-reactive protein favored in-hospital mortality. 

Because we studied a small group of hospitalized patients, our estimates are conservative and may not consider the general population or large numbers of infected patients. In addition, ECG has limited predictive power. Consequently, it is better to add advanced echocardiography and cardiac magnetic resonance imaging (imaging for myocardial tissue) to surface electrocardiography.

Our data demonstrated the important role of surface ECG in risk stratification of patients infected with COVID-19. Among the various electrical abnormalities, increased heart rate, ST-segment elevation, and atrial fibrillation are most strongly associated with outcome.
